# Wet Feet

**Published:** 1870-10

**Authors:** 


### WET FEET.

Take two parts of tallow and one of rosin, melt them together, stir the mixture well, dip the shoe in the hot mixture so as to nearly cover the sole, let it cool, dip it again, and repeat this process until the sole will absorb no more, or apply the mixture to the bottom of the sole repeatedly. If this is properly done, the sole will not only last longer, but will be impervious to dampness. As health is impaired, and even life lost sometimes, from damp feet, both economy and prudence dictate attention to this point.

